# Empagliflozin improves pressure-overload-induced cardiac hypertrophy by inhibiting the canonical Wnt/β-catenin signaling pathway

**DOI:** 10.3389/fphar.2024.1499542

**Published:** 2024-11-27

**Authors:** Xun Yuan, Li Pan, Chi Zhang, Qiulian Zhu, Zexin Huang, Yuan Qin, Guiping Zhang, Zhimei Feng, Caixian Yang, Ning Hou

**Affiliations:** ^1^ The Affiliated Qingyuan Hospital, Guangzhou Medical University, Qingyuan, China; ^2^ Department of Pharmacology, School of Pharmaceutical Sciences, Guangzhou Medical University, NMPA Key Laboratory for Clinical Research and Evaluation of Drug for Thoracic Diseases, Guangzhou Medical University, Guangdong Provincial Key Laboratory of Molecular Target and Clinical Pharmacology, Guangzhou Medical University, Guangzhou, China; ^3^ Department of Physiology, School of Basic Medicine Sciences, Guangzhou Health Science College, Guangzhou, China; ^4^ School of Stomatology, Guangzhou Medical University, Guangzhou, China

**Keywords:** empagliflozin, hypertrophy, FZD, Wnt, beta-Catenin

## Abstract

**Background:**

Empagliflozin (EMPA) is an SGLT-2 inhibitor that can control hyperglycemia. Clinical trials have indicated its cardio-protective effects against cardiac remodeling in diabetes or non-diabetes patients. However, the underlying molecular mechanisms of EMPA’s cardio-protective effects remain elusive.

**Methods:**

We evaluated whether the EMPA attenuated the pressure-overload-induced cardiac hypertrophy by inhibiting the Wnt/β-catenin pathway. Furthermore, the effects of the EMPA on a mouse model of transverse aortic constriction (TAC) induced cardiac hypertrophy was also evaluated. Mice were administrated with 0.5% CMC-Na as a vehicle or EMPA (10 mg/kg/day, daily, throughout the study) by intragastric gavage.

**Results:**

The *in vivo* echocardiography and histologic morphological analyses revealed that EMPA attenuated TAC-induced cardiac hypertrophy. Moreover, it also ameliorated TAC-induced cardiac fibrosis and decreased the cell size of the cardiomyocytes in isolated adult cardiomyocytes. Molecular mechanism analysis revealed that the EMPA reduced the TAC-induced enhanced expression of the Wnt/β-catenin pathway *in vivo*. For *in vitro* assessments, isolated neonatal rat cardiomyocytes (NRCMs) were treated with Angiotensin II (AngII) and EMPA; the results showed that in the absence of EMPA, the expression of the Wnt/β-catenin pathway was enhanced. In the trans-genetic heterozygous β-catenin deletion mice, EMPA attenuated TAC-induced cardiac remodeling by reducing the Wnt/β-catenin pathway. In addition, molecular docking analysis indicated that EMPA interacts with FZD4 to inhibit the TAC and AngII induced Wnt/β-catenin pathway in cardiomyocytes.

**Conclusion:**

Our study illustrated that EMPA might directly interact with FZD4 to inhibit the TAC and AngII-induced activation of the Wnt/β-catenin pathway to attenuate the adverse cardiac remodeling.

## 1 Introduction

Cardiac hypertrophy is an adaptive response of the heart to mechanical or neurohumoral stimulation (Brancaccio et al., 2003). Several studies have identified persistent pathological myocardial hypertrophy as an independent risk factor for heart failure (HF), sudden death, and fatal arrhythmias (Luo et al., 2021; Hu et al., 2022). Furthermore, it has been observed to be accompanied by increased interstitial collagen levels and myofibroblast activation, resulting in cardiomyocyte apoptosis, myocardial fibrosis, decreased myocardial contractility, and a further decline in cardiac function, which leads to fatal congestive HF (Luo et al., 2012; Santos-Gallego et al., 2021). Despite the significant therapeutic advances, cardiac hypertrophy remains irreversible (Byrne et al., 2017). Therefore, it is essential to comprehensively investigate the cellular mechanisms underlying cardiac hypertrophy. New molecular targets and innovative therapeutic strategies are needed to slow or reverse progressive cardiac hypertrophy.

Empagliflozin (EMPA) is a C-glucoside derivative that is based on the structure of the natural compound Phlorizin. It has been observed to selectively block sodium-glucose cotransporter 2 (SGLT2) on renal proximal tubule epithelial cell membrane, inhibit glucose reabsorption, and promote an insulin-independent hypoglycemic effect (Packer et al., 2020). Furthermore, it is considered a new class of hypoglycemic drug and has attracted much attention because of glucose-lowering and its unique cardiovascular protective effects. The EMPA-REGOUTCOME (Zinman et al., 2015) revealed that EMPA significantly reduced the hospitalization rate of HF and the relative risk of cardiovascular events in type 2 diabetes mellitus patients. Moreover, the EMPRISE (Patorno et al., 2019) study validated that EMPA can reduce the hospitalization risk of HF in type 2 diabetic patients with or without a history of cardiovascular disease (CVD). In addition, the EMPEROR-Reduced clinical trials indicated that EMPA also reduced the incidence of cardiovascular death and hospitalization in chronic HF patients with or without type 2 diabetes. The European Society of Cardiology recommends the use of EMPA for adult HF patients with or without diabetes and decreased ejection fraction to reduce the risk of cardiovascular death and hospitalization for HF (Ponikowski et al., 2016). The EMPEROR-Preserved trial showed that EMPA significantly reduced the risk of HF outcomes (Filippatos et al., 2022). It has been observed that for acute HF, EMPA intervention provides significant clinical benefit within 90 days after starting treatment (Voors et al., 2022). Long-term EMPA treatment in HF patients has indicated persistent benefits in chronic HF patients, which dissipated rapidly after the drug withdrawal (Packer et al., 2023). In addition, EMPA has also been observed to reduce the risk of HF in patients with cardiac dysfunction after acute myocardial infarction (Hernandez et al., 2024). Recent research advances in preclinical studies showed that EMPA improves oxidative phosphorylation and attenuates pressure overload HF (Li et al., 2021). Moreover, it attenuates HF with preserved ejection fraction via the Erbb4 pathway (Ni et al., 2024). However, the SGLT2 receptor has been reported as significantly renal specific and is only expressed in the renal cortex but not in the human heart. Therefore, how EMPA benefits the heart is undetermined as the specific mechanisms remain elusive.

The canonical Wnt/β-catenin signaling pathway has been observed to be essentially associated with cardiovascular development and CVDs. Furthermore, it has been reported to modulate gene expression during different pathophysiological conditions, such as myocardial hypertrophy, myocardial fibrosis, and HF. β-catenin is the core protein of the canonical Wnt signaling pathway (Nusse and Clevers, 2017; Ozhan and Weidinger, 2015).

The activated canonical Wnt/β-catenin signaling secretes Wnt, which binds to Frizzled (FZD) receptors and low-density lipoprotein-related receptor (LRP) co-receptors. CK1α and GSK3β then phosphorylate LRP receptors, which recruit Dishevelled (Dvl) proteins to the plasma membrane, resulting in the stabilization and accumulation of β-catenin. The stabilized β-catenin then trans-locates into the nucleus to form an active complex with lymphoid enhancer factor and T-cell factor proteins (LEF/TCF) to initiate gene transcription. The Wnt/β-catenin pathway reportedly has a pivotal role in cardiac hypertrophy and HF. Several studies have indicated that the β-catenin levels are increased in phenylephrine- or endothelin 1-induced cardiomyocyte hypertrophy. Furthermore, overexpressed β-catenin has been found to promote protein synthesis, nuclear polyploidy, and cell size increase (Foulquier et al., 2018). Whereas β-catenin knockdown in NRCMs markedly reduced the cell surface area. In addition, cardiac β-catenin knockdown alleviates the stress-induced cardiac hypertrophy (Iyer et al., 2018). Our previous study found that the hearts of patients with ischemic heart disease or idiopathic dilated cardiomyopathy had enhanced activity of the Wnt/β-catenin pathway and nuclear accumulation of β-catenin (Hou et al., 2016). However, how EMPA affects the Wnt/β-catenin pathway, specifically in cardiac hypertrophy, remains undetermined.

Therefore, this study investigated whether EMPA promotes its protective role by modulating the Wnt/β-catenin pathway and explored the underlying molecular mechanisms using a transverse aortic constriction (TAC)-induced cardiac hypertrophy mouse model and TAC model derived primary adult mouse cardiomyocytes (AMCMs). The findings will increase the current molecular understanding of EMPA-induced cardiac protective effects and may provide novel therapeutic targets to protect the heart from stress-induced cardiac dysfunction.

## 2 Materials and methods

### 2.1 Ethical approval

The study design was reviewed and approved by the Animal Ethics Committee of Guangzhou Medical University (Guangzhou, China) according to the Guidelines for Animal Care and Experimentation with approval number GY 2018-104.

### 2.2 Transverse aortic constriction procedure

Male C57BL/6J mice (age: 6–8 weeks, weight: 20 ± 2 g) were housed in a controlled environment and provided the free access to food and water. For TAC procedure, all the mice were anesthetized with isofluorane. Then, the midline sternotomy was performed, the thymus was separated, and the aortic arch was exposed. Then, a 6.0-silk suture was wrapped around the arch between the innominate artery and the left carotid artery over a 26-gauge needle. Subsequently, the needle was removed, and the chest was sutured. The sham group mice underwent all the procedures except the aortic constriction. For analgesia, buprenorphine (2 mg/kg) was administered thrice after surgical incision. After 7 days of surgery, 0.5% CMC-Na as the vehicle and EMPA (10 mg/kg/day) were administrated via oral gavage for the duration of the study.

### 2.3 Transgenic mouse model

Mice with two loxP sites inserted in the β-catenin gene’s 1 and 6 introns position were donated by Dr. Rolf Kemler (Max-Planck Institute of Immunology, Germany) (Brault et al., 2001) nd housed at The Jackson Laboratory (Stock No.004152) (Bar Harbor, ME). Transgenic mice expressing Cre recombinase under the control of the α-myosin heavy chain promoter (αMHC-Cre) were donated by Dr. M D Schneider and maintained at The Jackson Laboratory (Stock No.011038) (Agah et al., 1997). The homozygous β-catenin loxP-floxed (β-catenin flox/flox) mice were mated with α MHC-Cre mice to produce cardiac-specific β-catenin knockdown mouse models. Because conventional and cardiomyocyte-specific β-catenin -knock-out mice are embryonically lethal (Ye et al., 2015), the heterozygous β-catenin deletion Het (β-cat flox/+, αMHC-Cre+) mice or WT (β-cat flox/+, αMHC-Cre-) mice were subjected to TAC surgery for subsequent experiments. Littermate wild-type mice without Cre recombinase expression were used as controls. Supplementary Table S2 enlists all the genotyping primers utilized.

### 2.4 Echocardiography and Doppler imaging

Visual Sonics Vevo 2,100 system equipped with an MS-400 transducer (Visual Sonics, Inc.) was employed for echocardiography. Left ventricle ejection fraction (LVEF) and other systolic functions were obtained from the short-axis with M-mode scans, as indicated by the presence of papillary muscles. For anesthesia, 3% isoflurane was administered, and the lack of response to firm pressure under a body temperature-controlled pad indicated successful anesthesia. Isoflurane was reduced to 1.0%–1.5% and adjusted to maintain a heart rate between 350 and 550 (Beats per minute; BPM). The (Heart Rate; HR) (Left ventricular end-diastolic diameter; LVED; d), (Left ventricular end-systolic diameter; LVED; s) (Left ventricular internal systolic dimension; LVID; s, (Left ventricular internal diastolic dimension; LVID; d) (Left ventricular end-systolic anterior wall thickness; LVAW; s), (Left ventricular end-diastolic anterior wall thickness; LVAW; d (Left ventricular end-diastolic posterior wall thickness; LVPW; d), (Left ventricular end-systolic posterior wall thickness; LVPW; s) (Left ventricular fractional shortening; FS), and (Left ventricular ejection fraction; EF), were detected. All the mice recovered from anesthesia without difficulty. All parameter data were presented as a mean of at least 5x continuous image analysis.

### 2.5 Preparation for heart tissue samples

After the final Echocardiograph analysis, the mice were weighed, anesthetized using isoflurane, and sacrificed by cervical dislocation. The hearts were dissected in cold PBS and weighed before the Ventricle and Atrium were separated. The Ventricle was snap-frozen in dry ice and transferred into a −80°C refrigerator. Tibias were also removed.

### 2.6 Immunoblot analysis and immunoprecipitation

Heart tissue and cells were homogenized with RIPA lysis buffer (Thermo Fisher Scientific; Cat # 89901) containing halt protease and phosphatase inhibitor cocktail (Thermo Fisher Scientific; Cat # 78442), incubated in the shaker for 30 min at 4°C, and then centrifuged at 12,000 *g* (30 min) to collect supernatant for immunoblotting.

Whole lysate protein concentration was measured by Pierce BCA Protein Assay Kit (Thermo Fisher Scientific; Cat # 23225). Then, an equal quantity of protein lysates was isolated on 8%–15% SDS-PAGE gel by electrophoresis, transferred to 0.45 μm PVDF membranes, blocked in 5% non-fat milk diluted in TBST for 45 min at room temperature, probed overnight with primary antibodies diluted in a blocking buffer at 4°C on a shaker. Then, the membranes were treated for 45 min with an HRP-conjugated secondary antibody diluted in a blocking buffer at room temperature, and the target proteins were detected using SuperSignal West Pico Chemiluminescent Substrate (Thermo Fisher Scientific; Cat # 34580). Amersham Imager 600 was used for immunoblotting processing.

For immunoprecipitation, the extracts from the ventricular tissue were cleared using Protein A/G beads, and the mixtures were subsequently centrifuged at 1,000 *g* for 4 min at 4 °C. The supernatants were incubated with primary antibodies at 4 °C overnight. On the 2nd day protein A/G beads incubated for 4 h, and the beads were collected and washed three times with lysis buffer. After the final wash, the proteins were collected and subjected wo Western blot analysis using the antibodies listed in Supplementary Table S3.

### 2.7 Histology and imaging

The dissected mice hearts were put in ice-cold PBS, ventricle slice was acquired, fixed for 60 min in 4% paraformaldehyde in PBS, washed twice with cold PBS, placed in 30% sucrose in PBS for 30 min, embedded in optimal cutting temperature (OCT) compound, snap frozen in dry-ice, and stored at −80°C. The heart sections were cut into 5 μm slices using cryostat. The myocardial tissue sections were fixed, and the treated cardiomyocytes were washed with 0.1 mol/L glycine for 25 min to reduce the non-specific crosslinking activity of residual paraformaldehyde. The sections were then immersed in 10% normal goat serum for 60 min to block non-specific binding and incubated overnight at 4°C with Alexa Fluor 488 conjugated antibody for 1 h, followed by Alexa 550 conjugated α-actinin antibody for 1 h. The nucleus was stained with DAPI (Sigma-Aldrich, St. Louis, MO). Confocal images were collected with a Nikon A1R confocal upright microscope under uniform settings. From each mouse, 3 heart sections were utilized for this analysis to determine the percentage intensity of antibodies.

### 2.8 Isolation of adult mouse ventricular myocytes

Adult mouse cardiac left ventricular myocytes were isolated by following the method described previously (Chen et al., 2022). Briefly, the hearts were perfused with perfusion medium (Gibco) containing 1.2 mmol/L MgSO_4_, 10 mmol/L HEPES, 4.6 mmol/L NaHCO_3_, 30 mmol/L taurine, 10 mmol/L 2,3-butanedione monoxime, and 5.5 mmol/L glucose. After perfusion, the heart was digested with the isolation buffer using the Langendorff system with type II collagenase (Worthington, Cat # LS004174, NJ, United States). Then, forceps and pipettes were used to dissect and mechanically dissociate left ventricles (LV) in a perfusion medium. Isolated cells were fixed with 4% paraformaldehyde or stored at −80°C for the subsequent experiments.

### 2.9 Neonatal rat cardiomyocyte (NRCM) isolation, culture, and drug treatment

NRCMs were isolated from one to two-day-old Sprague Dawley rats, as described in our previous study (Chen et al., 2022). Briefly, the ventricles were homogenized using D-Hank’s balanced salt solution, trypsinized by 0.05% trypsin, followed by 0.2% collagenase (type II), and seeded into plates containing DMEM to remove fibroblasts and non-cardiomyocytes. The cells were cultured with DMEM augmented with 10% fetal bovine serum (FBS) and 0.1 mmol/L bromodeoxyuridine and then treated with vehicle alone (0.05% DMSO), Angiotensin II (AngII) (1 μM) + vehicle (0.05% DMSO), and AngII (1 μM) + EMPA (1, 5, 10 μM, respectively) for 24 h before collection.

### 2.10 Molecular docking

Molecular docking was performed to predict the possible binding of EMPA (EMPA) with FZD. The crystal structures of human FZD4, human FZD5, and mouse FZD8 were acquired from the protein data bank (PDB) under the following codes: 5CM4, 5URZ, and 1IJY, respectively. The reported potential binding sites were chosen as docking centers. Docking was performed using Surflex-Dock GeomX (SFXC) in SYBYL-X 2.1.1 software (Tripos, United States), and the results were evaluated by total score (unit: log10). The highest score was selected as the binding affinity in the best binding mode. The Discovery Studio Visualizer 2020 (Dassault Systems BIOVIA, United States) was utilized to demonstrate the two-dimensional diagrams of the ligand-receptor interactions.

### 2.11 293T cell culture, drug treatment, and FZDs plasmids transfection

HEK293T cells were cultured in DMEM (Thermo Fisher Scientific, Waltham, MA, United States) supplemented with 10% FBS (Biochrom, Berlin, Germany) without antibiotics. The full-length human FZD4, human FZD5, and human FZD8 were cloned into pcDNA3.1 vectors (Youbio, Hunan, China.) Lipofectamine^®^ 3,000 reagent (Invitrogen; Thermo Fisher Scientific, Inc., CA, United States) was used for transfecting the FZDs or negative control (NC) plasmids, per the kit’s instruction. After 16 h of transfection, the cells were treated with Wnt3a (100 ng/mL, R and D Systems, MN, United States), or Wnt3a + EMPA (50 mM) for 48 h before collection for subsequent analyses.

### 2.12 Luciferase reporter assay

Luciferase reporter assay was performed according to a standard protocol. HEK293T cells (1 ×10^6^/well) were cultured in 60 mm plates for 24 h and then transfected with plasmids (FZD4, FZD5, FZD8) and PC-DNA (5 μg/well) using Lipofectamine 3,000 reagent (Thermo Fisher Scientific, Waltham, MA, United States, Cat. No. L3000008). After 16 h of transfection, the culture medium was removed, and 5 mL/well of DMEM with 10% FBS containing Wnt3a (400 μg) and 50 mM EMPA was added for another 48 h. Then, luciferase and Renilla signals were determined by a Dual-Luciferase Reporter Assay Kit (V Cat. No. DL101-01; Vazyme, Nanjing, China) on a Wallac 1,420 Victor microplate reader (PerkinElmer, United States). The Firefly/Renilla signal ratios were recorded.

### 2.13 Statistical analysis

SPSS 18.0 and Prism GraphPad software were employed for all the statistical measurements, and the data are presented as the mean ± SEM. Statistical analyses included one-way ANOVA with a *post hoc* Tukey test, Two-way ANOVA with a *post hoc* Tukey test. Statistically, the *p*-value of <0.05 was deemed as the significance threshold.

## 3 Results

### 3.1 Effects of empagliflozin on biochemical indicators in TAC mice

In this study, a mouse cardiac hypertrophy model was established by surgical ligation of the transverse aortic. No significant differences were observed in the serum glucose level, total serum cholesterol (TC), triglyceride (TG), low-density lipoprotein (LDL), and renal function parameters among these four groups (Supplementary Table S1). Furthermore, high-density lipoprotein (HDL) was decreased, and glutamic oxaloacetic transaminase (AST) was increased in the TAC group, which was attenuated after EMPA treatment (*p* < 0.05, Supplementary Table S1).

### 3.2 Empagliflozin improved cardiac function and attenuated the TAC-induced cardiac remodeling

**FIGURE 1:**
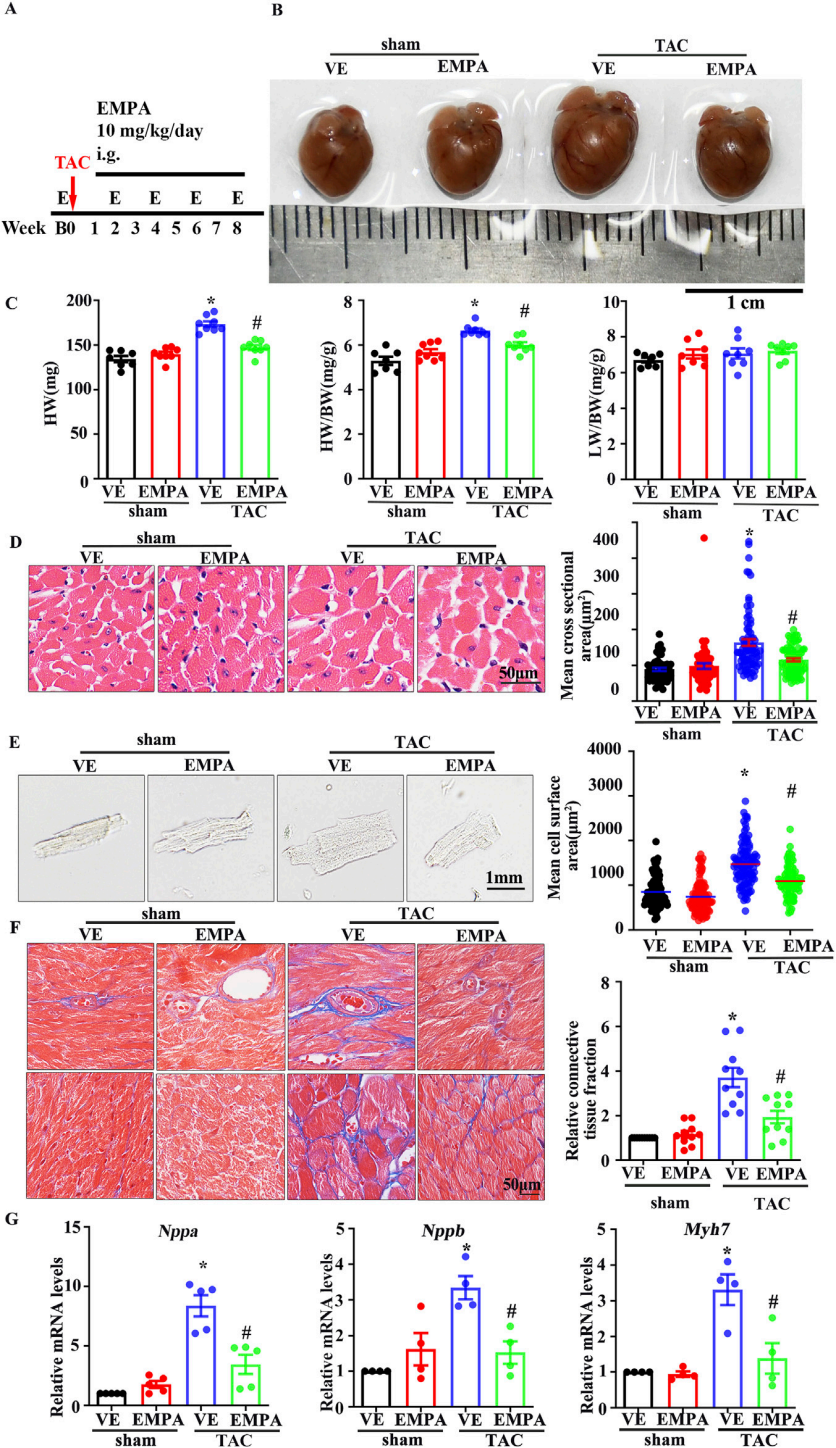
Empagliflozin attenuates TAC-induced cardiac hypertrophy **(A)** Schematic diagram of TAC-induced cardiac hypertrophy mouse model, EMPA administration, and Echocardiography testing **(B)** Representative heart images from Sham, Sham + EMPA, TAC + VE, TAC + EMPA at the ends of the experiments. **(C)** HW, HW/BW ratio, and dry LW/BW ratio in different groups (n = 6–8). **(D)** Cardiomyocyte sizes were measured by H&E staining (n = 6). **(E)** Immunofluorescence image representing the increased size of cardiomyocytes isolated from Sham, Sham + EMPA, TAC + VE, and TAC + EMPA groups. **(F)** Cardiac fibrosis was measured by Masson staining. The relative connective tissue fraction area was analyzed. **(G)** Relative transcripts of *Nppa, Nppb, and Myh7* in Sham, Sham + EMPA, TAC + VE, and TAC + EMPA groups. GAPDH was used for transcript normalization. Results are expressed as mean +SEM. *p < 0.05 vs. Sham + VE, #*p <* 0.05 vs. TAC + VE. One-way ANOVA was used to analyze each group. TAC: Transverse aortic constriction, EMPA: Empagliflozin, VE: Vehicle, HW: Dry heart weight, BW: Body weight, LW: Dry lung weight, H&E: Hematoxylin-eosin staining.

The effects of EMPA on TAC-induced myocardial hypertrophy and ventricular remodeling were also assessed. The 10 mg/kg/day EMPA was administered for 1 week after the surgery (Figure 1A), and it was observed that compared with the vehicle treatment, EMPA attenuated TAC-induced cardiac hypertrophy (Figure 1B), heart weight (HW)/body weight (BW) ratio, and lung ventricle weight (LW)/body weight (BW) ratio (Figure 1C). In addition, EMPA treatment significantly reduced the cardiomyocyte’s cross-sectional area in TAC mice (Figure 1D) compared to the mice in the sham group. Furthermore, the cardiomyocytes were isolated from each mouse’s heart to measure their surface area, which revealed that EMPA treatment decreased the size of individual cardiomyocytes (Figure 1E). Since myocardial fibrosis is a key feature of cardiac remodeling, Masson trichrome analysis was performed on cardiac tissue sections to evaluate the myocardial interstitial and perivascular fibrosis. The EMPA treatment reduced cardiac fibrosis in TAC mice compared to the sham mice (Figure 1F). Overall, continuous administration of EMPA for 8 weeks significantly attenuated the cardiac hypertrophy manifestations and fibrosis index (Figures 1B–G). Moreover, the mRNA levels of hypertrophic genes, *Nppa*, *Nppb*, and *Myh7* were upregulated in TAC mice’s hearts but significantly reduced in the EMPA-treated mice (Figure 1G).

**FIGURE 2:**
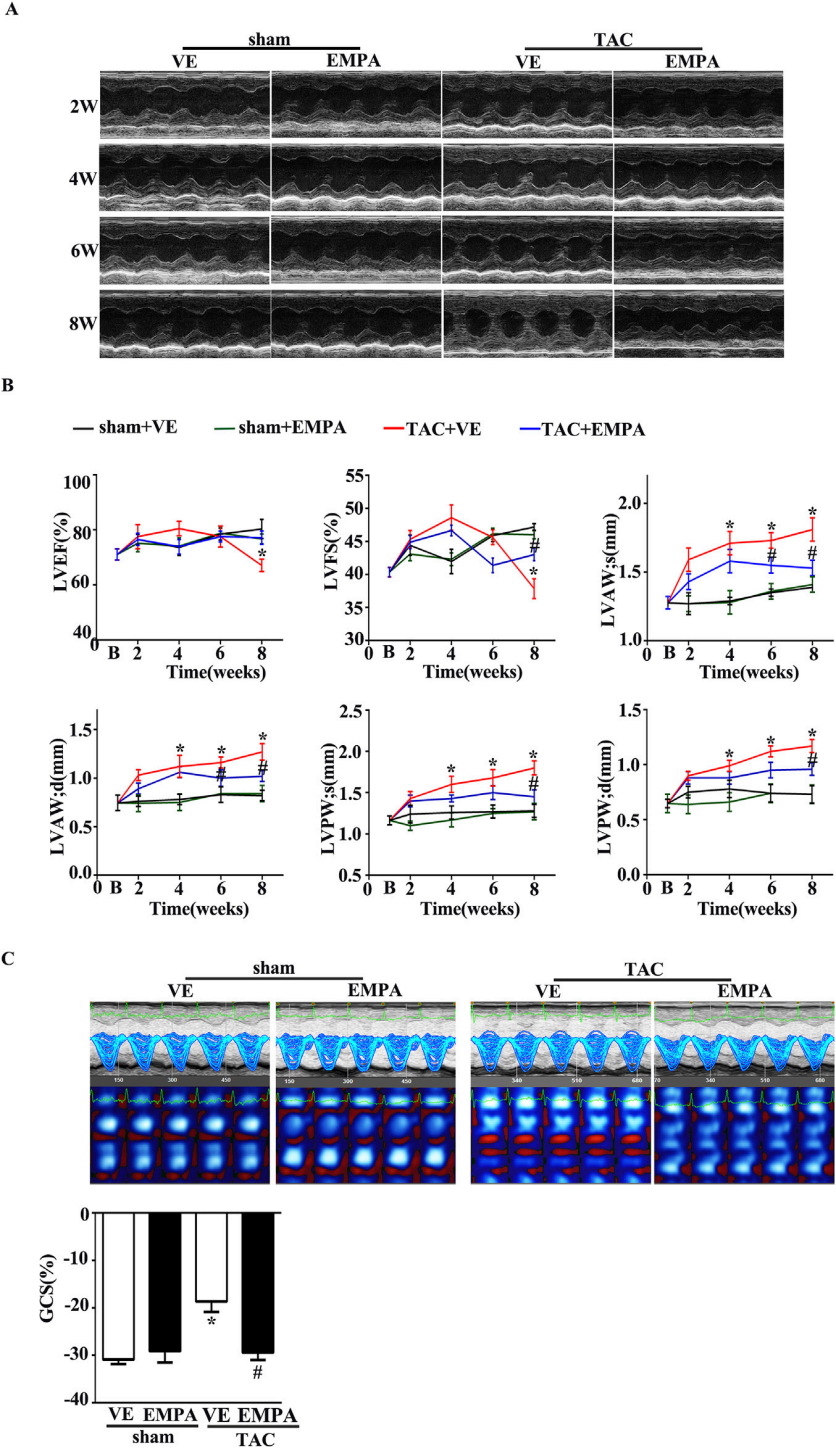
Empagliflozin treatment attenuated TAC-induced cardiac hypertrophy and delayed TAC-induced progressive cardiac systolic dysfunction. **(A)** Representative M-mode images from Sham, Sham + EMPA, TAC + VE, and TAC + EMPA at 2, 4, and 6 weeks and the end of the experiment. **(B)** Cardiac systolic function and wall thickness were quantified, including LVEF, LVFS, the end-diastole left ventricle posterior and anterior wall thickness, the end-systole left ventricle posterior and anterior wall thickness in different groups (n = 6–8). **(C)** Cardiac speckle-tracking analysis was performed to evaluate cardiac function. Representative GCS images and quantitative graph from Sham, Sham + EMPA, TAC + VE, and TAC + EMPA at the end of the experiments (n = 6). Results are expressed as mean +SEM. *p < 0.05 vs. Sham + VE, #*p <* 0.05 vs. TAC + VE. One-way ANOVA was used to analyze each group. TAC: Transverse aortic constriction, EMPA, Empagliflozin, VE: Vehicle, GCS: Global circumferential strain, LVEF: Left ventricle ejection fraction, LVFS: Left ventricle fraction shortening.

Echocardiography of each group of mice was performed every 2 weeks after EMPA administration to evaluate the function and structure of heart ventricles. Left-ventricular systolic function was indicated by LVEF and LV fractional shortening (LVFS). Compared to the sham group, LVEF and LVFS significantly increased from 2 weeks after TAC surgery and peaked at 4 weeks, which decreased 6 weeks thereafter (Figures 2A, B). Moreover, LVAW and LVPW at both systole and diastole in the TAC group increased gradually compared with the sham group. However, 4 weeks of EMPA treatment significantly improved the above echocardiographic parameters.

Recently, Speckle Tracking Echocardiography (STE) has emerged as an important diagnostic modality in cardiac diagnostic imaging. Global circumferential strain (GCS) from STE is a novel index of LV function and has been used to estimate LV filling pressures and predict outcomes in several disease states. Therefore, in this study, the LV systolic function was assessed with GCS from STE. Consistent with the changes of LVEF and LVFS, EMPA treatment markedly attenuated the TAC-induced LV dysfunction (Figure 2C).

### 3.3 Empagliflozin inhibited the activation of canonical Wnt/β-catenin pathway in TAC-induced cardiac hypertrophy

**FIGURE 3:**
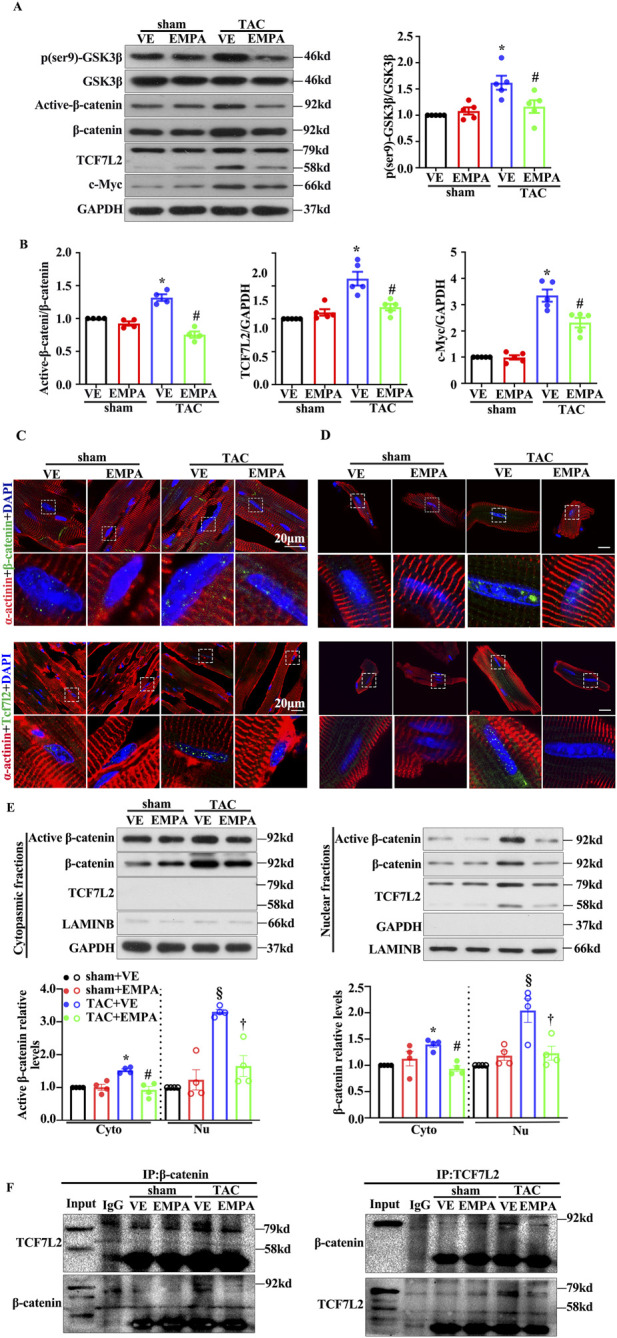
Empagliflozin treatment attenuated TAC-induced β-catenin/TCF7L2 axis activation. **(A)** Representative Western blots and relative protein levels of p-GSK3β, GSK3β, active-β-catenin, β-catenin, TCF7L2, c-myc, GAPDH in the heart of mice from indicated groups. **(B)** Quantitative results of the relative protein level of active-β-catenin, TCF7L2, c-myc are shown. **(C)** Representative immunofluorescence images of the level and location of β-catenin and TCF7L2 in the heart of mice from the indicated groups. Red = α-actinin, Green = β-catenin or TCF7L2, and Blue = DAPI. Cytoplasmic and nuclear extracts were isolated to assess the relative protein levels of active β-catenin, β-catenin, and TCF7L2 in the hearts of mice from the indicated groups. **(D)** Cytoplasmic and Nuclear fractions are isolated by the isolated buffers. **(E)** Representative Western blots of active β-catenin, β-catenin, TCF7L2, GAPDH, and LAMINB in the hearts of mice from the indicated groups. **(F)** Representative immunoprecipitation (IP) and Western blot (IB) images indicated the interaction between β-catenin and TCF7L2 in hearts obtained from the indicated groups. Endogenous β-catenin or TCF7L2 was purified by immunoprecipitation using anti-β-catenin or anti-TCF7L2 antibodies, respectively (input: 10% of lysate). Results are expressed as mean +SEM. *p < 0.05 vs. Sham + VE, #*p <* 0.05 vs. TAC + VE, §*p <* 0.05 vs. Sham + VE, †*p <* 0.05 vs. TAC + VE. One-way ANOVA was used to analyze each group. TAC: Transverse aortic constriction, EMPA, Empagliflozin, VE: Vehicle, Cyto: Cytoplasmic, Nu: Nucleu, IgG: Immunoglobulin G.

The canonical Wnt/β-catenin pathway has been observed to be associated with cardiac hypertrophy. Previous studies have indicated that cardiac β-catenin deletion can attenuate stress-induced cardiac hypertrophy within 4 weeks. To assess if EMPA can inhibit the canonical Wnt/β-catenin pathway in TAC mice, GSK3β phosphorylation levels and the protein expression of Non-phospho (active) β-catenin and total-β-catenin were identified by Western blotting using the heart tissues from each group. The phosphorylation levels of GSK3β′s Ser9 residue, a key regulator of cytoplasmic β-catenin level, as well as the Non-phospho (active) β-catenin level, were significantly increased in TAC mouse hearts compared with the vehicle (Figures 3A, B). These results were consistent with previous findings. Furthermore, TCF7L2, a key transcriptional factor in nuclei of β-catenin, increased dramatically. The Wnt/β-catenin pathway’s downstream target, c-Myc, was also observed to be notably expressed. However, the EMPA treatment markedly reduced the levels of GSK3β phosphorylation, active-β-catenin, TCF7L2, and c-Myc (Figures 3A, B). The landmark of canonical Wnt/β-catenin pathway activation is β-catenin nuclear accumulation. Therefore, immunofluorescence and cellular fractionation analyses were performed to analyze the β-catenin location. Immunofluorescence and Western blot analyses confirmed that both active- and total-β-catenin were accumulated in the nucleus of the TAC mice heart cells (Figures 3C, E). These changes were repressed by EMPA treatment (Figures 3C, E). Immunofluorescence assay in adult cardiomyocytes isolated from each group of mice hearts further validated these findings (Figure 3D). Moreover, the co-immunoprecipitation analysis revealed that TAC significantly promoted the β-catenin and TCF7L2 co-localization, whereas the EMPA treatment had opposite effects (Figure 3F). These data suggested that the inhibition of the canonical Wnt/β-catenin pathway may play a critical role in the improvement of cardiac hypertrophy by EMPA.

These data suggested that TAC-induced reduction of the β-catenin/TCF7L2 pathway might be associated with the targeted mechanism of EMPA, which reduces the primary composite endpoint of major adverse cardiovascular events, cardiovascular death, and hospitalization rate for HF, as observed in EMPA-REGOUTCOME trial.

### 3.4 Empagliflozin protected against AngII-induced cardiomyocyte hypertrophy by inhibiting the canonical Wnt/β-catenin pathway

**FIGURE 4:**
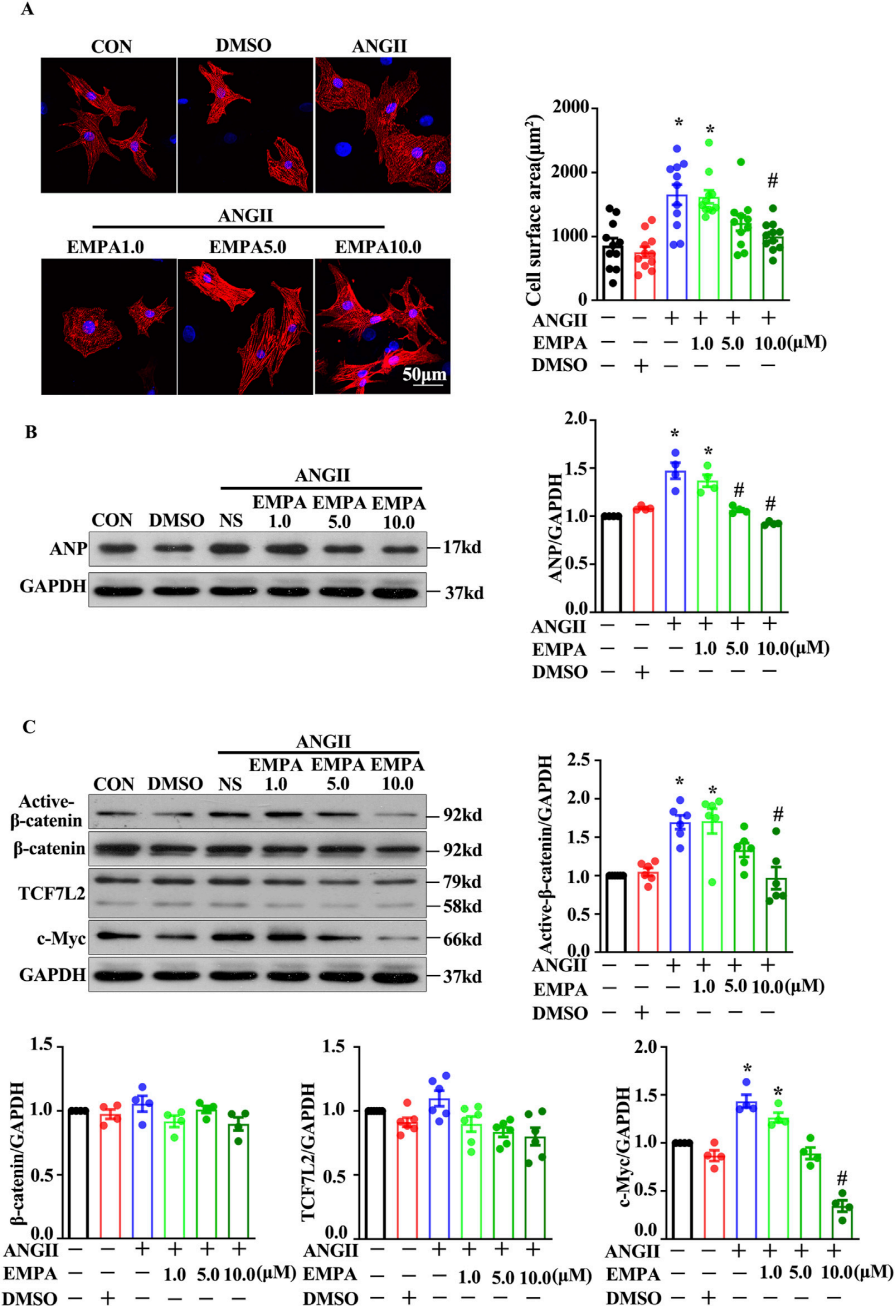
Empagliflozin treatment decreased the Wnt/β-catenin pathway in ANG2-treated neonatal rats cardiomyocytes **(A)** Representative immunofluorescence images showing the level and location of β-catenin and TCF7L2 in NRCMs stimulated with Ang-II with or without EMPA (48 h); Red = α-actinin, Green = β-catenin, and Blue = DAPI **(B)** Representative Western blots and relative protein levels of ANP. **(C)** Active β-catenin, β-catenin, TCF7L2, and c-myc in NRCMs stimulated with Ang-II with or without EMPA. Results are expressed as mean +SEM. *p < 0.05 vs. CON, #*p <* 0.05 vs. ANGII + NS, One-way ANOVA was used to analyze each group. CON: Control, ANGII: Angiotensin II, EMPA, Empagliflozin, DMSO: dimethyl sulfoxide.

An *in vitro* AngII treatment model was employed to assess whether EMPA reduced cardiomyocyte hypertrophy by inhibiting the canonical Wnt/β-catenin pathway. The results in NRCMs were consistent with the findings in mouse hearts. After 24 h of co-treatment, EMPA significantly blocked the AngII (1 μM)-induced cardiomyocyte hypertrophic manifestations, including increased cell surface area and ANP expression (Figures 4A, B). Furthermore, EMPA dose-dependently repressed the AngII (1 μM)-induced active-β-catenin and c-Myc protein levels in the Wnt/β-catenin signaling (Figure 4C). Moreover, EMPA treatment reduced TCF7L2 expression, but the decrease was not statistically significant (Figure 4C).

### 3.5 Empagliflozin reduces the β-catenin/TCF7L2 pathway to attenuate the TAC-induced cardiac hypertrophy

**FIGURE 5:**
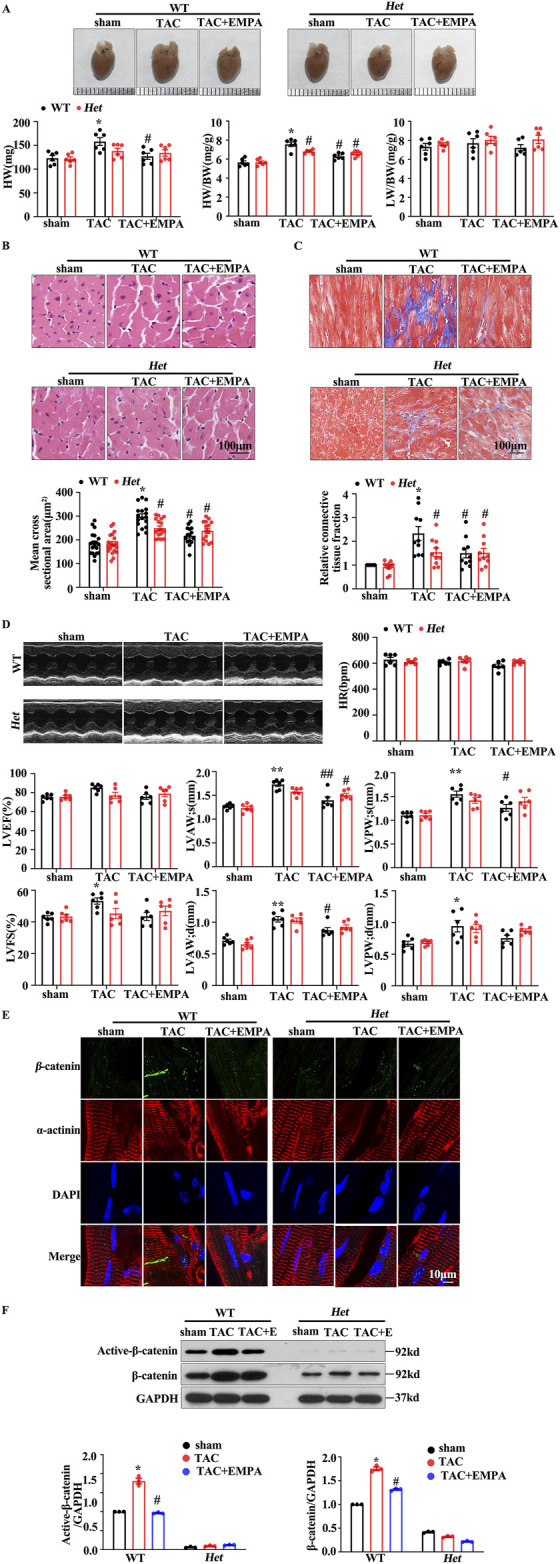
Empagliflozin reduced the β-catenin/TCF7L2 axis to delay the TAC-induced cardiac hypertrophy **(A)** Gross HW, HW/BW, and LW/BW values of the whole heart of the indicated groups **(B, C)** Representative images of H&E and Masson staining of heart tissues from the indicated groups. The mean cross-sectional area and relative connective tissue fraction in the indicated groups. **(D)** Echocardiographic analysis of cardiac function in TAC trans-genetic mice treated with or without EMPA. Representative M-mode images showing systolic cardiac function and quantification of parameters (LVFS) **(E)** Representative immunofluorescence images showing the level and location of β-catenin in the heart cells obtained from the indicated groups. Red = α-actinin, Green = β-catenin, and Blue = DAPI. **(F)** Representative Western blots and relative protein levels of active β-catenin and β-catenin in the heart tissues of the indicated groups. One-way ANOVA was used to analyze each group. Results are expressed as mean +SEM. *p < 0.05 vs. WT Sham, #*p <* 0.05 vs. WT TAC, Two-way ANOVA was used to analyze each group. TAC: Transverse aortic constriction, EMPA, Empagliflozin, DAPI: 4,6-diamino-2-phenyl indole, Het: Heterozygous, WT: Wild type.

To further investigate the role of β-catenin in TAC-induced cardiac pathological remodeling. Mice with cardiomyocytes-specific heterozygous deletion of the β-catenin coding gene; Het (β-catenin flox/+; α-MHC-Cre+) or WT (β-catenin flox/+, αMHC-Cre-) were stimulated with TAC and then treated with EMPA. The mice underwent TAC surgery or not before the NS or EMPA interventions. Compared to the wild-type (WT) mice, EMPA-treated mice had reduced TAC-induced cardiac hypertrophy (Figure 5A). Furthermore, Het mice indicated reduced levels of TAC-induced cardiac hypertrophy and diminished interstitial fibrosis (Figure 5B C and D). Het mice had decreased β-catenin protein expression in the heart (Figures 5E, F). Moreover, EMPA treatment inhibited the TAC-induced β-catenin/TCF7L2 axis activation (Figures 5E, F). These data suggest that EMPA reduces the TAC-induced β-catenin nuclei accumulation and its downstream transcriptional regulation to attenuate pathological cardiac remodeling. In summary, it was inferred that EMPA treatment attenuates the TAC-induced cardiac hypertrophy by reducing the β-catening/TCF7L2 pathway.

### 3.6 Molecular docking analysis of empagliflozin binding in frizzleds

**FIGURE 6:**
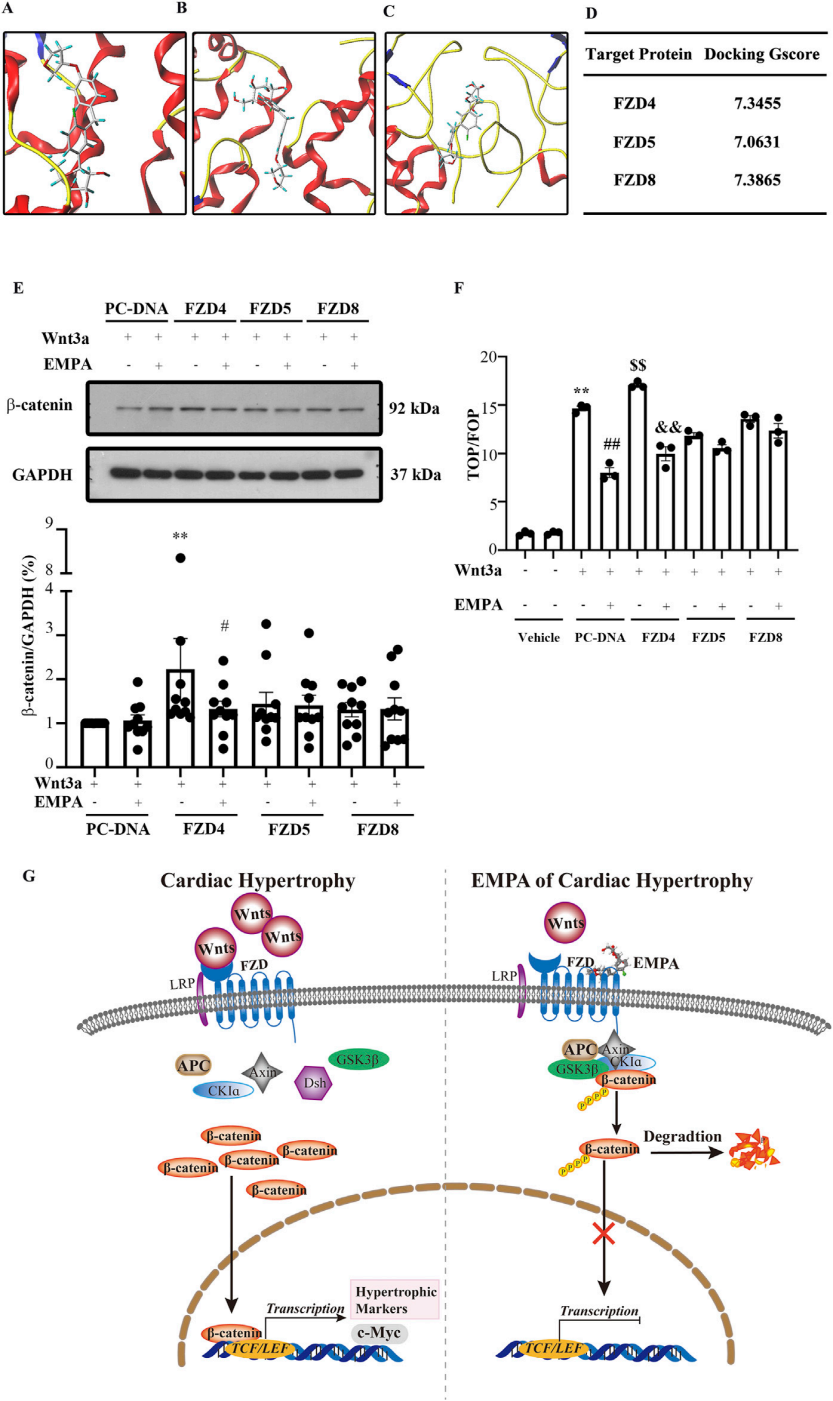
Empagliflozin directly binds Frizzled family members in the heart and inhibits the Wnt/β-catenin pathway. Molecular docking results showed that EMPA can bind **(A)** FZD4, **(B)** FZD5, and **(C)** FZD8. **(D)** The binding energy of each docking is shown. **(E)** Representative Western blots and the relative protein levels of β-catenin in the indicated groups' heart tissues. **p < 0.01 vs. PC-DNA + EMPA + Wnt3a, #*p <* 0.05 vs. FZD4 + Wnt3a. **(F)** The activity of TCF/β-catenin reporter in HET293T cells was analyzed by TOP/FOP FLASH. **p < 0.05 vs. BSA group, ##*p <* 0.01 vs. Wnt3a, $$ *p <* 0.01 vs. Wnt3A, && *p <* 0.01 vs. FZD4 + Wnt3a. One-way ANOVA was used to analyze each group. **(G)** Graphical abstract indicates that EMPA binds FZD4 to inhibit the Wnt/β-catenin pathway, thereby attenuating the TAC-induced cardiac hypertrophy. FZD: Frizzled family members, EMPA, Empagliflozin

The above results showed the inhibitory effect of EMPA on the Wnt/β-catenin pathway. However, the mechanisms underlying this inhibitory effect remain poorly understood. The Wnt/β-catenin pathway is activated when Wnt, FZD, and LRP5/6 form a complex, which stabilizes the β-catenin. A cysteine-rich domain (CRD) is in the extracellular region of FZD proteins, which has been implicated as the Wnt binding domain. Wnt binding to CRD of FZD activates the Wnt/β-catenin pathway. Therefore, in this study, molecular docking analysis was performed to predict the binding mode and affinity of EMPA with the CRD of FZD. Docking results showed that EMPA had a high affinity with FZD4 (binding score = 7.3455), FZD5 (binding score = 7.0631), and FZD8 (binding score = 7.3865) (Figures 6A–D). Furthermore, the best binding modes and affinity were observed when EMPA was docked into the middle of the FZD dimer. EMPA could bind to both mouse and human FZDs. Based on these data, it was hypothesized that EMPA inhibited the Wnt/β-catenin pathway via FZDs. To verify if EMPA directly binds to FZD, HEK293T cells were stably transfected with FZD-4, -5, and -8 plasmids, respectively. The cells were then stimulated with Wnt3a, in the presence of EMPA or without EMPA. The EMPA and Wnt3a co-treatment significantly inhibited the expression of β-catenin protein, which was further upregulated by Wnt3a treatment in FZD4-transfected cells, but EMPA treatment suppressed the β-catenin upregulation (Figure 6E). However, when FZD-5 and -8 transfected cells were treated with Wnt3a, no changes in the expression of β-catenin were observed.

This study also assessed the transcriptional activity of β-catenin via the TOP/FOP dual-luciferase reporter gene system, and the results were consistent with Figure 6E. When FZD4 transfected 293T cells were treated with Wnt3a, β-catenin transcriptional activity was increased, whereas EMPA treatment significantly inhibited this increase. Moreover, FZD-5 and -8 transfected cells treated with Wnt3a indicated no significant effect on the transcriptional activity of β-catenin. The luciferase reporter assay results indicated that EMPA inhibits FZD4-mediated TOPFLASH luciferase activity (Figure 6F).

The Wnt/β-catenin pathway is involved in the pathogenesis of cardiac diseases, especially cardiac hypertrophy. This study revealed that EMPA treatment can alleviate TAC-induced cardiac hypertrophy by directly inhibiting the FZD4/Wnt/β-catenin/TCF7L2 pathway in cardiomyocytes (Figure 6G).

## 4 Discussion

This study indicated that (1) EMPA inhibits the Wnt/β-catenin pathway in cardiomyocytes to attenuate TAC-induced cardiac hypertrophy. (2) Molecular docking revealed that EMPA binds FZD, which binds and activates Wnt. Altogether, EMPA inhibited the FZD/Wnt/β-catenin pathway activation in TAC-induced cardiac hypertrophy, thereby improving functional recovery and delaying cardiac hypertrophy.

Recent clinical trials have demonstrated that EMPA protects the hearts from nondiabetic HF and reduces ejection fraction (Packer et al., 2021; Santos-Gallego et al., 2021). However, little is known about the comprehensive mechanisms underlying the protective effect of EMPA against nondiabetic HF and reduced ejection fraction. This study utilized the TAC-induced pressure overload rodent model that recapitulates progressive cardiac hypertrophy and HF to observe cardiac hypertrophy and fibrosis tissues are like heart tissue obtained from hypertension patients. This model revealed that the EMPA treatment protects the heart from TAC-induced cardiac hypertrophy. Wnt/β-catenin/TCF7L2 pathway plays an important role in TAC-induced cardiac hypertrophy (Iyer et al., 2018). Furthermore, it was observed that inhibiting the Wnt/β-catenin/TCF7L2 pathway markedly improves cardiac structure and function. In summary, EMPA treatment protects the heart from non-diabetic cardiac hypertrophy and HF by reducing the Wnt/β-catenin/TCF7L2 pathway.

Several studies have indicated that EMPA’s protective effect on the heart is not directly related to the hypoglycemic effect. In the case of reduced ejection fraction, EMPA has been observed to show a protective effect on non-diabetic HF. Furthermore, it can effectively inhibit myocardial remodeling as well as improve cardiac function (Santos-Gallego et al., 2021) and the survival rate of patients. EMPA can reduce glycolysis by directly binding Glucose transporter 1 (GLUT1), and attenuate cardiac remodeling induced by pressure overload by increasing AMPK activity and reducing mTORC1 expression. Jiang et al. confirmed that in the myocardial cell membrane, EMPA can directly inhibit the activity of sodium/hydrogen exchanger 1 (the Na+/H+ exchanger1, NHE1), suppress excessive autophagy, improve ventricular remodeling, and decrease cardiac function after myocardial infarction (Li et al., 2021).

Several molecular mechanisms underlying the beneficial effects of EMPA in HF and doxorubicin-induced cardiac dysfunction have been reported, such as attenuation of glycolysis and AMPK-mTORC1 pathway and inhibition of cardiac inflammation (Sabatino et al., 2020; Li et al., 2021; Byrne et al., 2017; Mustroph et al., 2018; Koyani et al., 2020; Kolijn et al., 2021; Vaziri et al., 2023). However, clinical trials have indicated that the EMPA’s beneficial effects are independent of the cardiac energy metabolism or circulating serum metabolites associated with energy metabolism (Hundertmark et al., 2023). Previous studies have proved the EMPA-induced beneficial effects were independent of its antihyperglycemic effect (Santos-Gallego et al., 2021; Li et al., 2021). Since SGLT2 receptors are not present in the heart, EMPA treatment’s beneficial effects on the heart may be independent of SGLT2 inhibition. These data imply that the EMPA has direct effects on the heart. This study demonstrated that the EMPA suppresses the FZD/Wnt/β-catenin pathway to attenuate the TAC-induced cardiac hypertrophy.

It has been reported that EMPA significantly lowers the risk of HF and improves cardiac function in nondiabetic HF patients (Santos-Gallego et al., 2021). TAC and AngII administration are widely used methods for establishing a mouse cardiac hypertrophy model (Hu et al., 2004; Nakamura et al., 1998; Iyer et al., 2018). This study utilized an *in vivo* TAC-induced cardiac hypertrophy mouse model and *in vitro* AngII-stimulated hypertrophy in primary NRCMs to illustrate the benefits of EMPA on cardiac hypertrophy. EMPA promotes antihyperglycemic effects by increasing glycosuria and natriuresis in diabetic humans (Kovacs et al., 2014). In addition, the EMPA treatment did not alter the blood glucose in non-diabetic mice (Li et al., 2021). Studies have indicated that speckle-tracking echocardiography can more sensitively predict early cardiac dysfunction (Kang et al., 2014; Li et al., 2021). To evaluate EMPA’s effects on TAC-induced cardiac dysfunction, GLS and GCS of the LV were evaluated. EMPA significantly increased the GCS in TAC-induced mice. These suggested that EMPA significantly attenuates cardiac function without hypoglycemia. The literature has indicated that the Wnt/β-catenin pathway is activated in CVDs and human HF (Iyer et al., 2018; Hou et al., 2016; Duan et al., 2012; Fu et al., 2019). Consistently, here, it was observed that EMPA treatment corrected the cardiac activation of the Wnt/β-catenin/TCF7L2 pathway, reversing the TAC-induced cardiac hypertrophy. Overall, these data revealed an EMPA alleviates cardiac and individual cardiomyocyte hypertrophy by interfering with Wnt/β-catenin/TCF7L2 signaling in the cardiomyocytes.

Since SGLT2 receptors are not present in the heart, molecular docking was employed to predict the potential binding target of the EMPA in the heart, which revealed that the EMPA interacted with Wnt membrane receptor FZD4 (Gscore 7.3455), FZD5 (Gscore 7.0631), and FZD8 (Gscore 7.3865) with high affinity. Furthermore, the pharmacological data on recombinant FZD validated that SGLT-i EMPA is a potent FZD inhibitor. Moreover, AngII was employed to induce cardiomyocyte hypertrophy, which was alleviated by EMPA. Altogether, EMPA attenuated the TAC-induced cardiac hypertrophy.

Wnt3a reportedly stimulates WNT signaling (MacDonald et al., 2009). Which has been positively associated with cardiomyocyte proliferation. Activation of WNT/β-catenin signaling by deletion of APC led to ventricular hyperplasia (Ye et al., 2015). Furthermore, reduced cardiac WNT protein expression alleviated adverse cardiac remodeling and strengthened cardiac function at 30 days post-myocardial infarction (Palevski et al., 2017). FZD proteins are seven transmembrane receptors, which comprise seven hydrophobic amino acid helices allowing them to embed in a membranous structure. FZD family members are G-protein coupled receptors and encode proteins accountable for cell signal transduction, proliferation, and death. Wnts interact with FZDs via their CRD at the N terminus, thereby activating a distinct downstream signaling pathway (Liu et al., 2024).

It has been observed that FZDs are associated with the pathogenesis of various diseases, such as cancer, cardiac hypertrophy, familial exudative vitreoretinopathy, etc. Among the 10 members of FZDs, the biological role of the FZD4 gene has been most comprehensively investigated. It is highly expressed in cardiomyocytes and is involved in cardiac development and diseases (van Gijn et al., 2002). Yoon et al. (2018) found that FZD4 could serve as an index for lateral plate mesoderm, which increased cardiomyocyte proliferation via the canonical Wnt pathway. Recently, Yin et al. (2021) established the acute myocardial infarction model by ligation of the left anterior descending branch and found that FZD4 mediated the β-catenin/NF-kB signaling to promote cardiac fibrosis. These data indicated that the FZD4 is a key target for inhibiting the canonical Wnt/β-catenin pathway. Therefore, it was inferred that EMPA might interact with FZD4 to inhibit the TAC and AngII induced Wnt/β-catenin pathway in cardiomyocytes.

Several clinical trials and pre-clinical studies have indicated that EMPA is a promising therapeutic drug for non-diabetic HF patients. Its protective effect is potentially SGLT2 independent, implying that other molecular mechanisms underlie EMPA’s cardiac benefits. This study found that oral EMPA treatment decreases the TAC-induced activation of the Wnt/β-catenin/TCF7L2 signaling, thereby significantly attenuating LV hypertrophy. Furthermore, the molecular docking analysis revealed that the EMPA inhibits the Wnt/β-catenin/TCF7L2 signaling by competitive binding to the FZD protein. Therefore, EMPA may attenuate TAC-induced cardiac hypertrophy by binding FZD membrane protein, thereby inhibiting the Wnt/β-catenin/TCF7L2 signaling. Moreover, the β-catenin trans-genetic mice were employed to manifest the Wnt/β-catenin/TCF7L2 signaling involves the therapeutic mechanisms. The clinical trials have also reported that EMPA intervention promotes significant cardiac therapeutic effects. Therefore, this study suggests that the Wnt/β-catenin/TCF7L2 signaling plays a critical role in EMPA-induced attenuation of TAC-induced cardiac hypertrophy.

This research demonstrated that EMPA attenuates the progressive TAC-induced cardiac hypertrophy by directly binding to FZD to inhibit the Wnt/β-catenin/TCF7L2 signaling in cardiomyocytes, thereby protecting the heart from progressive HF. Although this is a pre-clinical experiment study, clinical trials have reported that the EMPA treatment protects hearts from non-diabetic mellitus CVDs. These findings highlighted potential therapeutic molecular mechanisms of the EMPA treatment. However, since the Wnt/β-catenin/TCF7L2 signaling is activated in diseased hearts, further pre-clinical studies on different cardiac-diseased models are warranted.

## Data Availability

The original contributions presented in the study are included in the article/Supplementary Material, further inquiries can be directed to the corresponding authors.

## Abbreviations

EMPA, Empagliflozin; SGLT2, Sodium-dependent glucose transporters 2; VE, Vehicle; TAC, Transverse aortic constriction; FZD, Frizzled; LRP, Lipoprotein-related receptor; NRCM, Neonatal rat cardiomyocytes; LEF, Lymphoid enhancer factor; TCF, T-cell factor proteins; AMCM, Adult mouse cardiomyocytes.
