# Supplementary material for: Empagliflozin improves pressure-overload-induced cardiac hypertrophy by inhibiting the canonical Wnt/β-catenin signaling pathway
Source: Front Pharmacol. 2024 Nov 27;15:1499542. doi: 10.3389/fphar.2024.1499542 (PMC11631586; doi:10.3389/fphar.2024.1499542)
Supplement: Supplementary file 1 [file DataSheet1.PDF]

Supplementary Material

1 Supplementary Tables

Table S1. Serum and biochemical indicators of TAC mice treated with vehicle or EMPA for 8 weeks

	Sham+VE (n=5)	sham+EMPA (n=5)	TAC+VE (n=5)	TAC+EMPA (n=5)
GLU(mmol/L)	4.04±0.38	3.74±0.45	4.07±0.27	3.30±0.46
Na^{+}(mmol/L)	152.00±0.41	151.25±0.25	150.83±0.54	151.28±0.73
Ca^{2+}(mmol/L)	2.19±0.02	2.23±0.02	2.23±0.02	2.19±0.03
TG(mmol/L)	1.06±0.18	1.15±0.12	1.08±0.09	1.19±0.18
TC(mmol/L)	2.92±0.14	3.08±0.19	2.69±0.21	2.78±0.12
LDL-C(mmol/L)	0.13±0.02	0.17±0.02	0.18±0.02	0.15±0.02
HDL-C(mmol/L)	2.44±0.09	2.48±0.12	2.00±0.14*	2.45±0.05#
UA(umol/L)	116.00±31.76	123.75±28.85	122.17±32.82	129.14±35.21
BUN(mmol/L)	11.21±0.60	11.99±0.79	12.09±0.81	12.18±0.55
Scr(umol/L)	13.50±0.87	13.25±1.60	13.17±0.65	13.43±0.62
AST(U/L)	103.00±2.65	119.55±6.61	162.22±10.14*	126.43±6.90
ALT(U/L)	44.00±5.43	40.75±7.97	53.50±5.16*	47.00±1.43

Table S2. Primer list

Target genes	Sequences (5'-3')
Nppa	Forward: CGGCTTCCTGCCTTCATCTATCAC<br>Reverse: GCGTCTGTCCTTGGTGCTGAAG
Nppb	Forward: AAGGCAGAGGCACCGTTGTTG<br>Reverse: GACAGCACCTTCAGGAGATCCATG
Myh6	Forward: ACTCCTCTTCCTGCCTGTTCCTC<br>Reverse: GGCGTCCGTCATTCTGTCACTC
Myh7	Forward: GCAAGACGGTGACTGTGAAGGAG<br>Reverse: GGTTGACGGTGACGCAGAAGAG
Myc	Forward: TAA CTC GAG GAG GAG CTG GA<br>Reverse: GCC AAG GTT GTG AGG TTA GG
Col1	Forward: AAGTCTCAAGATGGTGGCCG<br>Reverse: TCTCCGCTCTTCCAGTCAGA
Col3	Forward: AGAAAGGGGTCCTAGTGGCT<br>Reverse: TCCATCATTGCCTGGTCCAC
Tgfb1	Forward: AACCGCACTGTCATTCACCA<br>Reverse: AAACCGACCTTTGCCAATGC

Gapdh	Forward: AGGTCGGTGTGAACGGATTTG<br>Reverse: GGGGTCGTTGATGGCAACA

Table S3. Antibody list

Antibody Name	Cat Number	Application and dilution
Active β-catenin (92kd)	CST19807	WB(1:1000)
β-catenin (92kd)<br>β-catenin	CST8480<br>CST9582	WB(1:2000)<br>IF(1:200)
p(ser9) GSK3β (46kd)	CST9322	WB(1:5000)
GSK3β(46kd)	CST12456	WB(1:5000)
TCF7L2 (58,79kd)	CST2569	WB(1:1000)
TCF7L2	Ab76151	IF(1:400)
c-Myc (66kd)	CST18583	WB(1:500)
Cyclin D2 (31kd)	CST9582	WB(1:1000)
Lamin B (60kd)	AP6001	WB(1:5000)
α-actinin	BM0003	IF（1:200）
